# Supplementary material for: Left out: The effect of handedness on fMRI activation in memory paradigms
Source: Imaging Neurosci (Camb). 2026 Nov 3;4:IMAG.a.1441. doi: 10.1162/IMAG.a.1441 (PMC13732489; doi:10.1162/IMAG.a.1441)
Supplement: Supplementary file 1 [file IMAG.a.1441_supp.pdf]

Supplemental Table 1: fMRI Activations

Face Encoding: LH

Laterality	Region	Direction	#Voxels	X	Y	Z	p-val
B	Precuneus	-	11825	-1	-61	38	<0.001
R	Visual Stream	+	8770	36	-64	-7	<0.001
L	Visual Stream	+	7027	-36	-73	-8	<0.001
L	Angular Gyrus	-	4516	-56	-46	29	<0.001
R	Angular Gyrus	-	3216	55	-45	36	<0.001
R	DLPFC	-	2071	33	46	26	<0.001
L	DLPFC	-	1962	-35	45	25	<0.001
B	MPFC	-	931	0	35	20	<0.02
R	Superior Temporal Gyrus	-	600	62	-17	-1	<0.03
L	Amygdala	+	536	-23	-5	-18	<0.04
R	Inferior Frontal Sulcus	+	530	47	12	30	<0.04
B	VMPFC	+	464	-2	42	-17	<0.05

Face Encoding: RH

Laterality	Region	Direction	#Voxels	X	Y	Z	p-val
R	Visual Stream	+	13408	34	-65	-1	<0.001
L	Visual Stream	+	7391	-35	-75	-4	<0.001
R	Angular Gyrus	-	6028	56	-33	23	<0.001
B	Precuneus	-	5773	1	-52	42	<0.001
R	DLPFC/MPFC	-	5508	22	40	25	<0.001
L	Angular Gyrus	-	2585	-57	-48	33	<0.001
R	Inferior Frontal Sulcus	+	1667	49	22	22	<0.01
L	DLPFC	-	1292	-34	42	31	<0.01
L	Superior Temporal Gyrus	-	776	-48	3	-1	<0.02
R	Superior Frontal Gyrus	-	770	20	13	61	<0.02
L	Amygdala	+	641	-23	-3	-19	<0.03
B	VMPFC	+	587	0	39	-17	<0.03

Word Encoding: LH

Laterality	Region	Direction	#Voxels	X	Y	Z	p-val
B	Precuneus	-	4475	0	-56	38	<0.001
R	Angular Gyrus	-	2609	55	-51	27	<0.001
L	Inferior Frontal Gyrus	+	1494	-48	30	3	<0.01
L	Angular Gyrus	-	937	-58	-46	36	<0.02

Word Encoding: RH

Laterality	Region	Direction	#Voxels	X	Y	Z	p-val
B	Precuneus	-	2743	2	-58	38	<0.001
B	DMPFC	+	1913	-5	27	48	<0.01
R	Angular Gyrus	-	1761	57	-47	25	<0.01
L	Inferior Frontal Gyrus	+	1114	-45	31	-8	<0.01
L	Inferior Frontal Sulcus	+	979	-50	19	25	<0.02
R	Lateral Occipital	+	782	29	-92	-2	<0.02
L	Angular Gyrus	-	732	-58	-44	36	<0.03

Face Retrieval: LH

Laterality	Region	Direction	#Voxels	X	Y	Z	p-val
B	Dorsal Visual	+	7992	0	-64	42	<0.001
L	Ventral Visual	+	5083	-23	-78	-12	<0.001
L	Inferior Frontal Sulcus	+	2063	-45	15	34	<0.01
B	VMPFC	-	1962	2	44	-2	<0.01
B	DMPFC	+	1151	-3	25	48	<0.02
L	Anterior Insula	+	1038	-38	23	0	<0.02
R	DLPFC	-	907	22	37	47	<0.02
L	Frontopolar	+	904	-38	54	3	<0.02
R	Inferior Frontal Sulcus	+	685	42	15	29	<0.04
L	Caudate Nucleus	+	684	-10	10	7	<0.04
R	Caudate Nucleus	+	668	10	9	10	<0.04
R	Middle Temporal Gyrus	-	659	57	-4	-28	<0.04
R	Primary Visual Cortex	-	587	10	-76	-6	<0.04
R	Anterior Insula	+	529	35	26	-2	<0.05

Face Retrieval: RH

Laterality	Region	Direction	#Voxels	X	Y	Z	p-val
B	Dorsal/Ventral Visual	+	23671	-6	-66	19	<0.001
L	DLPFC/IFS/Anterior Insula	+	7683	-43	24	20	<0.001
B	Caudate Nucleus	+	2765	-1	5	8	<0.001
L	DMPFC	+	1885	-4	28	45	<0.01
R	Inferior Frontal Sulcus	+	957	41	15	33	<0.02
R	Anterior Insula	+	539	34	25	-4	<0.05

Word Retrieval: LH

Laterality	Region	Direction	#Voxels	X	Y	Z	p-val
B	Precuneus/Cingulate/Lateral Temporal/Medial and Lateral Frontal	+	67896	-4	-20	27	<0.001
L	Primary Visual/Cerebellum	+	9436	-6	-76	-24	<0.001
R	Primary Visual	-	1499	16	-81	-8	<0.01
L	Precentral	-	775	-45	-24	56	<0.02
L	Lateral Occipital	-	732	-23	-99	4	<0.02
R	Precentral	+	525	25	-31	61	<0.04
R	Anterior Insula	+	487	34	16	-16	<0.05

Word Retrieval: RH

Laterality	Region	Direction	#Voxels	X	Y	Z	p-val
B	Precuneus/Cingulate/Lateral Temporal/Medial and Lateral Frontal	+	82947	-3	-28	24	<0.001
R	Primary Visual	-	1213	16	-82	-9	<0.01
R	Anterior Insula/Inferior Frontal	-	778	42	13	-1	<0.02
L	Precentral	-	680	-42	-24	62	<0.03

Supplementary Table 2: MTL Anatomical ROI ANOVA Results

task	effect	num Df	den Df	MSE	F	ges	Pr(>F)
FaceEncoding	group	1	107	0.028	0.301	0.001	0.584
	hemisphere	1	107	0.007	9.805	0.008	**0.002**
	group:hemisphere	1	107	0.007	0.772	0.001	0.382
	ROI	1.90	203.51	0.013	5.124	0.015	**0.008**
	group:ROI	1.90	203.51	0.013	0.658	0.002	0.511
	hemisphere:ROI	1.36	145.55	0.015	1.155	0.003	0.301
	group:hemisphere:ROI	1.36	145.55	0.015	0.164	0.000	0.763
WordEncoding	group	1	107	0.126	0.177	0.001	0.675
	hemisphere	1	107	0.028	1.430	0.001	0.234
	group:hemisphere	1	107	0.028	1.924	0.002	0.168
	ROI	1.86	199.27	0.057	3.731	0.011	**0.028**
	group:ROI	1.86	199.27	0.057	2.377	0.007	0.099
	hemisphere:ROI	1.60	171.40	0.040	0.102	0.000	0.861
	group:hemisphere:ROI	1.60	171.40	0.040	1.171	0.002	0.304
FaceRetrieval	group	1	101	0.157	0.167	0.001	0.684
	hemisphere	1	101	0.020	1.549	0.001	0.216
	group:hemisphere	1	101	0.020	2.543	0.002	0.114
	ROI	1.98	199.61	0.047	6.861	0.019	**0.001**
	group:ROI	1.98	199.61	0.047	0.340	0.001	0.710
	hemisphere:ROI	1.49	150.53	0.033	2.242	0.003	0.124
	group:hemisphere:ROI	1.49	150.53	0.033	0.787	0.001	0.423
WordRetrieval	group	1	104	0.121	0.207	0.001	0.650
	hemisphere	1	104	0.020	2.377	0.002	0.126
	group:hemisphere	1	104	0.020	0.300	0.000	0.585
	ROI	1.85	192.38	0.049	3.179	0.010	**0.048**
	group:ROI	1.85	192.38	0.049	0.419	0.001	0.643
	hemisphere:ROI	1.51	157.01	0.033	1.474	0.002	0.233
	group:hemisphere:ROI	1.51	157.01	0.033	0.606	0.001	0.502
